# Matrix Metalloproteinases in Dental and Periodontal Tissues and Their Current Inhibitors: Developmental, Degradational and Pathological Aspects

**DOI:** 10.3390/ijms23168929

**Published:** 2022-08-11

**Authors:** Moataz Elgezawi, Rasha Haridy, Khalid Almas, Moamen A. Abdalla, Omar Omar, Hatem Abuohashish, Abeer Elembaby, Uta Christine Wölfle, Yasir Siddiqui, Dalia Kaisarly

**Affiliations:** 1 Department of Restorative Dental Sciences, College of Dentistry, Imam Abdulrahman Bin Faisal University, P.O. Box 1982, Dammam 31441, Saudi Arabia; 2 Department of Clinical Dental Sciences, College of Dentistry, Princess Nourah bint Abdulrahman University, P.O. Box 84428, Riyadh 11671, Saudi Arabia; 3 Department of Preventive Dental Sciences, College of Dentistry, Imam Abdulrahman Bin Faisal University, P.O. Box 1982, Dammam 31441, Saudi Arabia; 4 Department of Substitutive Dental Sciences, College of Dentistry, Imam Abdulrahman Bin Faisal University, P.O. Box 1982, Dammam, 31441, Saudi Arabia; 5 Department of Biomedical Dental Sciences, College of Dentistry, Imam Abdulrahman Bin Faisal University, P.O. Box 1982, Dammam 31441, Saudi Arabia; 6 Department of Conservative Dentistry and Periodontology, University Hospital, LMU Munich, Goethe Str. 70, 80336 Munich, Germany; 7 Department of Periodontology, Women Medical and Dental College, Abbottabad 22010, Pakistan

**Keywords:** matrix metalloproteinase (MMP), tissue inhibitors of metalloproteinases (TIMPs), dental tissues, periodontium, degradation dentistry, biomarkers, orthodontic movement

## Abstract

Objectives: This review article aims to describe some of the roles of Matrix metalloproteinases (MMPs) in enamel, dentine, dental caries, hybrid layer degradation, pulp and periodontal tissues, throwing light on their current inhibitors. The article addresses the potential of MMPs to serve as biomarkers with diagnostic and therapeutic value. Design: The sections of this review discuss MMPs’ involvement in developmental, remodeling, degradational and turnover aspects of dental and periodontal tissues as well as their signals in the pathogenesis, progress of different lesions and wound healing of these tissues. The literature was searched for original research articles, review articles and theses. The literature search was conducted in PubMed and MEDLINE for articles published in the last 20 years. Results: 119 published papers, two textbooks and two doctoral theses were selected for preparing the current review. Conclusions: MMPs are significant proteases, of evident contribution in dental and periapical tissue development, health and disease processes, with promising potential for use as diagnostic and prognostic disease biomarkers. Continuing understanding of their role in pathogenesis and progress of different dental, periapical and periodontal lesions, as well as in dentine-pulp wound healing could be a keystone to future diagnostic and therapeutic regimens.

## 1. Introduction

Peptidase is a term used to describe proteolytic enzymes that degrade peptide bonds in protein molecules and actively contribute to various developmental, physiological and pathological processes in animals and humans. They include serine, cysteine, aspartic, N-terminal threonine peptidases, glutamate peptidases, asparagine peptidases and metallopeptidases. Peptidases are divided into exopeptidases and endopeptidases. While endopeptidases identify certain amino acids in the middle of the protein molecule, exopeptidases target terminal amino acids in the peptide chain of a protein [1,2].

Matrix metalloproteinases (MMPs) compose a group of more than 25 secreted or cell surface linked calcium-dependent zinc-containing endopeptidases, which play an important role in the dynamics of the extracellular matrix through specific disassembling activities of extracellular matrix proteins and receptors. Although extracellular matrix is basically composed of water, proteins and polysaccharides, it has a tissue-specific unique composition that crucially determines the biochemical and biomechanical characteristics of each respective tissue. The extracellular matrix actively mediates the different intercellular adhesion and other biofunctional activities and changes with age [3,4,5,6,7,8]. MMPs are present in different animal species and humans [5,8], have different substrates, while possessing similar microstructural features [9]. Although the active site in MMPs involves metal ions, predominately zinc, it sometimes includes other metals like cobalt, manganese or nickel [1].

In general, MMPs contain a pre-domain, defining secretion or membrane insertion, a prodomain acting as an internal regulator by occupying the zinc in the following active site. The main active zinc-containing catalytic site includes the highly conserved histidine-rich consensus sequence [4,10,11], and is linked via a hinging sequence with the hemopexin domain, which defines substrate specificity [10]. Together with other proteases, MMPs and the natural endogenous (TIMPs) regulate the extracellular matrix, specifically the synthesis [9], re-modelling and degradation of extracellular matrix and pericellular substrates including proteinase, clotting factors, chemotactic substances, latent growth factors, receptors, cell membrane receptors and intercellular adhesive molecules [4,5,6,7,8,12]. They therefore actively and dynamically contribute during developmental, physiological, and pathological processes in different tissues [3,4,5,8]. The role of MMPs and TIMPs in regulating cellular behavior via tightly controlled proteolytic processes includes various developmental, physiological, and pathological aspects as well as disease resolution and healing phases [13].

MMPs are secreted in the extracellular matrix as inactive proenzymes or zymogens that are activated via complex biochemical processes to be responsible for proteolysis of specific parts of the extracellular matrix depending on the subtype [6]. MMPs can be activated by proteases, by physicochemical processes such as low pH or heat, and are controlled by their TIMPs [9]. MMPs are classified according to their presumed target substrate specificity, structure and biofunctionality, into five main classes: collagenases, gelatinases, stromelysins, matrilysins and membrane-type MMPs in addition to others. Although the developmental, physiological, and pathological roles of MMPs are evident, their exact mechanism of action is not fully elucidated [4]. MMPs are generally activated in the extracellular matrix or at the cell membrane but some can be activated intracellularly [4]. Their activation and interaction are regulated by certain extracellular matrix constituents as well as by TIMPs. Activation of MMPs occurs by cleavage of their NH2 terminal. Chemical interactions of signaling molecules, cytokines, growth factors or other MMP family members or by mechanical changes in the extracellular matrix can activate MMPs [14]. MMP inhibitors operate by substituting zinc ions or chelating calcium at the active terminal or interacting with zymogens before activation, or by coating the target substrate.

MMPs crucially contribute to all phases of tooth development, differentiation, growth, shaping, apoptosis and degradation of different dental and periodontal tissues. MMPs have become important as biomarkers of diseases including degradation of different dental tissues, reversible and irreversible pulpitis and apical periodontitis, as well as gingival and periodontal lesions [4,15,16,17,18,19,20,21,22].

This article reviews the role of MMPs in the development, remodeling and turnover of dental and periapical tissues and their signals in the pathogenic progress of different lesions affecting these tissues. The literature search was conducted in PubMed and MEDLINE for articles published in WoS and/or Scopus indexed journals after the year 2000, with one exception, that were selected according to relevance and significance and were used in the current literature review. A total of 119 published papers (original research articles, review articles), two textbook and two doctoral theses were selected for preparing the current review.

## 2. MMPs Grouping and Substrate

Based on respective substrate preference, domain structure and sequential similarity, MMPs are grouped into collagenases, gelatinases, stromelysins, matrilysins, membrane-type MMPs, and other MMPs [9].

**Figure 1:**
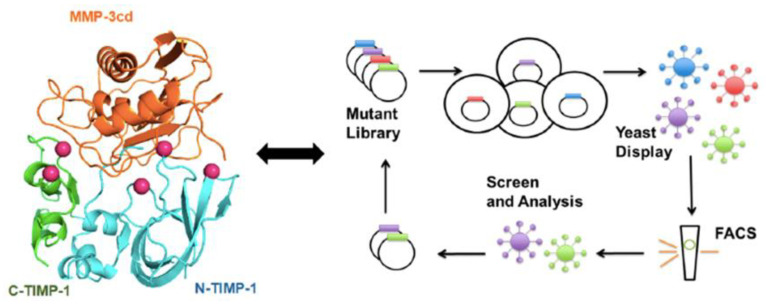
Engineering and designing MMPs inhibitors (from Raeeszadeh-Sarmazdeh, M.; Do, L.D.; Hritz, B.G. Metalloproteinases and Their Inhibitors: Potential for the Development of New Therapeutics. *Cells* **2020**, *9*, 1313. https://doi.org/10.3390/cells9051313, MDPI open access publication rules) [12].

Collagenases degrade certain extracellular matrix proteins, but they mainly cleave collagen type I, II, III, VII, X, gelatin, entactin, aggrecan, tenascin. This group contains MMPs-1, -8, -13 with similar action, while MMP-18 belongs to this group with limited action against type I collagen. Gelatinases decompose gelatin, collagen type I, IV, V, VII, X, XI in addition to elastin, fibronectin, laminin, aggrecan, vitronectin. Both MMP-2 and MMP-9 fit into this group and work on the previous substrates, but MMP-9 works on decorin, plasminogen and proTNF-α. Stromelysins have similar domain structure to collagenases, but they do not cleave interstitial collagen. MMP-3, and 10 by working on types III, IV, V, IX, X, XI collagens, proteoglycans, laminin, fibronectin, gelatin, aggrecan, elastin, fibrin/fibrinogen, vitronectin, while MMPs-3 has extended action on perlecan, decorin, proIL-1bc, plasminogen, Ecadherin, α2Ma, proTNF-α and MMPs-11 exist in this group without a recognized substrate. Matrilysins work mainly on fibrinogen, fibronectin, type IV collagen, gelatin, and laminin. Both MMP-7 and MMP-26 as members of matrilysins have this common action, but MMP-7 has a more extended range of action on collagen type I, III, V, IX, X, XI, tenascin, proteoglycans, pro α-defensin, vitronectin, proTNF-α, elastin, plasminogen, E-cadherin and decorin. Membrane-type MMPs have their cleavage effect commonly on gelatin and fibronectin, with a variable effect on laminin, perlecan, factor XII, tenascin, aggrecan, nidogen, entactin aggrecan, fibrin, vitronectin, proTNF-ab, transglutaminase, types I, II, III collagens, cartilage proteoglycan core protein, and α2Ma. The membrane-type MMPs have the subtypes MMP-14, -15, -16, -17, -24 and -25. Other minor MMPs were reported; MMP-12 as macrophage elastase has a similarity in its action to MMP-3 except for its limited action on collagen (collagen I, IV only). MMP-19 also bears some similarity to MMP-13 with limited action on collagen (collagen I, IV only) besides targeting casein, laminin, nidogen and nascin-C. MMP-20 with specificity in enamel by working on amelogenin, with some action on casein, gelatin, fibronectin, types IV, XVIII collagens, laminin, tenascin C and aggrecan. Some MMPs have not yet had a substrate determined, namely MMP-21 (xenopus) and MMP-23 (transmembrane type II). MMP-27 has action directed toward gelatin, collagen type II, fibronectin, while MMP-28 has activity against casein [9,23,24,25,26]. Modern therapeutic strategies are designing and engineering inhibitors of MMPs in promising endeavors to control and treat diseases [12], Figure 1.

## 3. MMPs and Enamel

Enamel is the hardest human tissue of the body that forms the external layer of the crowns of teeth; built by ameloblasts, the enamel forming cells are of ectodermal origin. While mature enamel is formed of up to 96 wt% inorganic minerals, 3% of the composition is water and less than 1% enamel proteins—at the secretory stage of enamel development matrix proteins, mainly amelogenin, form more than 30% of enamel by providing a scaffold for mineral growth. Proteases, mainly MMP-20 (enamelysin), with the substrates collagen V, aggrecan, amelogenin [10,27] and kallikrein-related peptidase 4 (KLK4) undergo cleavage of supporting enamel proteins, providing a space for the growth of crystalline minerals. This selective proteolysis of enamel proteins is the major regulator of the tooth’s external shape and quality of enamel. MMP-20 is expressed by ameloblasts early in the secretory phase until the early phase of enamel maturation, and shares in the cleavage of amelogenin, enamelin, and ameloblastin. At the bell stage of tooth development, MMP-20 degrades the basement membrane that exists between ameloblasts and odontoblasts facilitating their direct interactions. Accordingly, MMP-20 and KLK4 mutations can result in enamel developmental defects like amelogenesis imperfecta, clinically characterized by pigmented, soft, rough pitted enamel [28]. Broad spectrum drugs inhibiting effects on MMPs induce disturbances in enamel and dentine formation and mineralization [10]. Additionally, the genetic polymorphism rs478927 in MMP-13 is associated with caries occurrence and developmental defects of enamel in children from the Amazon region in Brazil [29].

MMP-2 (gelatinase), specific for denatured collagens: I, II, III, IV, V, VII, X, XI, aggrecan, elastin, fibronectin, gelatin, laminin, proteoglycan, MMP-9, MMP-13 [10], has additionally been detected in vivo in mouse models during the mucosal penetration stage of tooth eruption in the region of the lamina propria [30]. Gomes et al. (2010) conducted a study about the role of MMPs in the odontogenic region of the adult rat incisor tooth under different eruption conditions (normofunctional and hypofunctional). In the hypofunctional group, the authors found a relationship between the increase in eruption rate and the level of metalloproteinase bound to the cell membrane. MT1-MMP and TIMP-2 may have a role in cell proliferation during the eruption of the rat incisor tooth [31].

## 4. MMPs and Dentine

Dentine is the second hardest tissue in the human body forming the main core of the hard tooth structure. It consists of inorganic minerals, about 65 wt%, and 35 wt% organic matrix: including in detail 90% type I, III and V collagens as well as 10% non-collagenous proteins, for example dentine–sialo–phospho–protein and water [18,32]. These scaffolding matrix proteins enable nucleation and growth of the crystalline minerals of dentine together with mineral precipitation and cell-derived matrix vesicle mineralization [33]. Odontoblasts, of mesenchymal origin, migrate pulpally during dentinogenesis, laying down dentine matrix proteins and synthesizing, amongst others, MMPs for the regulation and remodelling of dentine matrix during dentinogenesis. The principal MMPs distinguished in pulp, predentine and dentine of sound non-carious teeth includes MMP-8 (collagenase), MMP-2 (gelatinase), MMP-9, MMP-13, MMP-14, and MMP-20 [4,28,34].

In sound dentine, MMP-2 is expressed most prevalently and progressively increases with the beginning of dentinogenesis. MMP-2 plays a major role in degradation of the basement membrane between ameloblasts and odontoblasts, allowing direct contact which is essential for further differentiation [34]. MMP-9 plays a significant role in dentine remodeling by targeting the dentine sialoprotein [34,35]. Later in dentinogenesis, MMP-2 and MMP-9 are located near the dentinoenamel junction, in association with increased gelatinase activity in mantle dentine. MMP-2 and MMP-20 contribute to the extracellular matrix formation. MMP-2 is also found in mature dentine and plays a role in caries pathogenesis, MMP-3 is seen in predentine and contributes to dentine mineralization [34]. Recently, it has been demonstrated using electron microscopy and reverse zymography analysis, that in sound human dentine TIMP1 is closely related to MMP-2 and MMP-9 and can bind to different isoforms of MMPs [36].

In addition to their presence in predentine, MMPs are detected extensively near the dentinoenamel junction, which might explain the wider lateral spread of caries as they penetrate the dentinoenamel junction. They were also detected in the dentinal fluid in the dentinal tubules [4]. MMPs are involved in mature and secondary dentine formation and mineralization in sound teeth, matrix degradation in dentine lesions, tertiary dentine formation and pulp inflammation. Tumor growth factor expressed by mature odontoblasts has a downgrading role on MMP-8, which might influence reparative dentine formation [4].

## 5. MMPs and Dental Caries

Caries are a multifactorial process, whereby according to the ecological plaque hypothesis, an imbalance of oral microflora—normally more than 700 different species—leads to an increase in the cariogenic bacteria mainly Streptococcus mutans and Lactobacillus types. The accumulating cariogenic bacteria produce acids like lactic acid that reduce the local pH, leading first to the demineralization and later to the destruction of the organic matrix through the activation of endogenous MMPs in saliva, gingival fluid, and dentine [37,38]. Caries progress as demineralization cycles prevail and remineralization cycles cease [39].

Classically, bacterial proteases are blamed for the proteolytic process taking place because of dental caries. However, activated endogenous MMPs in dentine, gingival crevicular fluid and saliva, share in degrading the dentine matrix of demineralized dentine at neutralized pH levels. Buffering of the saliva occurrs since MMPs operate only in neutral pH values [4,14]. Moreover, activated endogenous MMPs and cystine cathepsins participate in dentine matrix degradation in dental caries, in addition to bacterial proteases [40]. Collagen in the caries affected dentine retains the capability to remineralize until it is totally devoid of mineral nanocrystals [14]. Modern biomimetic approaches have achieved success in remineralizing completely demineralized dentine matrix [18,41].

Bacterial collagenases in addition to endogenous MMPs of salivary, gingival fluid and dentinal origin share in the dentine matrix degradation process in active carious lesions. The endogenous dormant MMPs are activated by local pH changes indicating the contribution of bacterial acids. The comparatively higher levels of MMP-8 and -9 in the outer zones relative to the inner caries affected zones indicate the role of MMPs of salivary origin in the process. In addition to salivary MMPs, cystine cathepsins are identified in saliva with potential collagenolytic activity in dental caries. Although it is not currently feasible to fully elucidate the role of MMPs of salivary, dentinal, or pulpal origin (through the dentinal fluid) in the process of dental caries, different reports support the contribution of dentinal fluid as an origin of MMPs and cathepsins in dental caries. MMP-2 may play a role in the lateral spread of caries beneath the dentinoenamel junction in early caries since it occurs in higher levels in the outer versus inner caries layers [42]. Moreover, significant gelatinolytic activities are seen in dentinal tubules with gelatinases showing a granular appearance near the pulp and not towards the dentinoenamel junction. This might indicate bulking of MMPs in matrix vesicles and their dentinal tubular transfer [22].

An enzyme linked immunosorbent assay of dentinal fluid collected from both shallow and deep carious lesions found significant correlation between MMP-9 in shallow and deep caries. These findings indicate that individuals with more MMP-9 in deep caries are likely to have more MMP-9 in shallow caries [43]. Higher levels of MMP-1 and -2 are found in the saliva of patients with caries rather than in healthy individuals. However, the levels of MMP-1 and -2 decrease after treatment [19].

MMP-1 (collagenase-1), MMP-2 and -9 (gelatinase-A and -B), MMP-3 (stromolysin-1), MMP-8 (collagenase-2) and MMP-20 (collagenase-3) are currently known to participate in dental caries and dental restoration failure [42]. During the carious process, pro-MMPs become activated through acidic pH (4.5). Following their activation, MMPs become stable by pH neutralization due to the salivary buffering effect [42].

Animal studies show that certain chemicals with MMP inhibitors such as modified tetracycline and zoledronate, are effective in reducing dentine caries, which demonstrates the significance of MMPs in dental caries [14,44]. During dental caries, MMPs develop proteolytic activity: MMP-20, MMP-2, -3, -9 and -8 are detected in carious dentine in dormant and active forms. In detail, MMP-1 and especially MMP-8 work as collagenases, the most powerful digesting type I collagen. MMP-2 and MMP-9 gelatinases, have the potential to disrupt the C-terminal of the collagen molecule. However, while MMP-9 is identified in greater concentrations in deep levels of caries, MMP-2 has no variation regarding caries depth [14,43]. MMP-3 releases proteoglycans like decorin, followed by cytokines that potentiate degradation of the demineralized dentine matrix [34]. Cysteine cathepsin of dentine is able to activate latent MMPs. At increasing depth of carious lesion, cathepsin activity becomes stronger with greater collagenolytic potential as more MMPs become activated [45]. Regardless of the rate of progress of dental caries, endogenous dentine MMPs decrease with aging [4]. TIMPs (1, 2, 3, 4) potentiate the proteolysis of dentine matrix during the carious process through an imbalance with MMPs [46]. Dentine degradonomics is a modern approach introduced in caries research to identify proteases and their substrates in different physiological and pathological processes of dentine using genomic and proteomic technologies. In this regard, genetic encoding, and definition polymorphism analysis of MMPs are performed in different studies to identify the association with dental caries in different populations [22,47]. Investigating the genetic association of MMP-10, MMP-14, and MMP-16 with dental caries shows that MMP-16 SNP rs2046315 is associated with dental caries [48].

The evident role of MMPs in the pathogenesis and progress of dental caries has drawn the interest of researchers to stop dental caries, not only by combating cariogenic microorganisms, but also by developing inhibitors for endogenous MMPs in dentine and saliva in the form of gels and mouth washes and stop caries and promote healing and remineralization [46]. Moreover, MMP inhibitors are suggested to resist dentine abrasion and erosion [49].

Fluorides have long been used in dentistry to prevent caries and induce inhibitory effects of MMPs. Sodium fluoride and stannous fluoride inhibit salivary and purified human gelatinases, MMP-2 and MMP-9. Treatment of dentine with sodium trimetaphosphate, a synthetic compound that reduces dentine demineralization, inhibits MMP-2 and MMP-9 activities particularly at 1.5% concentration [50,51]. Silver diamine fluoride, in addition to antibacterial and collagen remineralizing effects, has an inhibiting effect on MMPs that increases the degradation resistance of demineralized collagen to the proteolytic activities of MMP-2, -8 and -9, and inhibits the collagenolytic action of cysteine cathepsin but leaves unaesthetic black stains [52]. Although the exact mechanism of inhibition of MMPs by fluorides is not fully elucidated, fluorides, due their high electronegativity, might bind to zinc and calcium cations essential for the effectiveness of MMPs [53]. Identification of the peptide products of dentine matrix degeneration by MMPs in the carious process might have potential therapeutic value in dentine and bone regeneration strategies [34]. Chlorohexidine, ethylene-diamine-tetra-acetic-acid and chemically modified tetracyclines are among the MMP inhibitors for controlling dental caries [22].

## 6. MMPs in Pulpal and Periapical Lesions

Odontoblasts and fibroblasts of the pulp can also express MMPs, especially MMP-13 and MMP-1 [4,54]. In reversible and irreversible pulpitis, MMPs play a bifunctional role of tissue destruction and downgrading, together with tissue protection and mediation of host immune responses [4,55]. During progression of caries, proteolytic cleavage of dentine matrix by MMP-1, -3, -8, -9, -13 and more significantly MMP-20, can play a signaling inductive dentinogenesis for tertiary dentine formation and dentine-pulp wound healing [56]. Although the application of MMP-3 could induce regeneration in rat teeth with injured pulps and in teeth with irreversible pulpitis, it fails to produce similar valid effects in human clinical trials [21].

On the other hand, there is a more increased release of active MMPs in pulpitis than in healthy pulp tissue, indicating their role in pulp inflammation: released cytokines (IL-1β) and tumor necrosis factor-α (TNF-α) in pulp inflammation, activate MMP-1, MMP-2 and TIMP1 gene expression [45]. While MMP-2 expression was observed in the dental papilla cells, dental follicle, ameloblasts, odontoblasts and bone cells from the coronal and basal regions of the bony crypt [30], bacteroids and anaerobic bacteria can also stimulate excretion of MMP-1, MMP-2 and TIMP1 by the pulp cells [45]. The level of MMP-2 in root canal exudate of teeth with pulp necrosis or asymptomatic apical periodontitis is reduced gradually with root canal treatment procedures, which might validate MMP-2 as a biomarker [57].

Higher levels of MMP-8 are found in irreversible pulpitis with higher pain scores [58,59], explicitly expressed by polymorphonuclear leukocytes, macrophages, plasma cells and some endothelial cells of the blood vessels of the pulp tissue proper, suggesting the role of MMP-8 in extracellular matrix degradation during pulp and periapical tissue inflammation. The level of MMP-8 progressively decreases after 15 days of a mineral trioxide aggregate (MTA) pulpotomy procedure in rat molars, significantly more than Biodentine and calcium hydroxide pulpotomies, indicating the superiority of MTA for vital pulp therapy [60].

MMP-9 expression is enhanced in inflamed pulps, especially in endothelial cells, inflammatory infiltrate, odontoblasts, and fibroblasts [61]. In patients with symptomatic irreversible pulpitis treated with a single visit mineral trioxide aggregate pulpotomy, active MMP-9 concentration in pulpal blood has a significant correlation with the outcome, possibly indicating a prognostic biomarker [62].

In a recent clinical study, inflammatory cytokines and MMPs were assessed in collected dentinal fluid after selective caries removal and treating dentine with self-etching adhesives in patients with deep caries. They were used in immunoassays as biomarkers of inflammation to detect the influence of clinical procedures of selective caries removal and adhesive materials on the pulp tissue. Eight weeks following selective caries removal, MMP-8 and TIMP1 levels increase [63].

In an animal study, MMP-9 and MMP-2 have a strong correlation with progression of apical periodontitis [64]. Melatonin and 5-methoxytryptophol are effective in reducing MMP-1 and -2 levels in the serum and pulp tissue of acute pulpitis models in rats pointing to future potential therapeutic measures [65].

Bone resorption in apical periodontitis is linked to host inflammation and immune response. As osteoclasts start their bone resorption activity, MMPs such as MMP-9 should be functional since they contribute to degradation of the bone organic matrix. Biomarkers of bone resorption in apical periodontitis including MMP-9 in controlled diabetic and normoglycemic patients are not significantly different [66]. In patients with apical periodontitis presented clinically by a negative sensibility test and apical radiolucency, MMP-9 is reduced by sodium hypochlorite and sodium hypochlorite limewater. When an intracanal medication of calcium hydroxide and chlorohexidine is used, reduction in MMP-9 and MMP-8 levels is potentiated [67].

## 7. MMPs and Hybrid Layer Degradation

Resin bonding to dentine is a routine practice in restorative dentistry for direct and indirect restorations [68]. The procedure involves either etch-and-rinse or self-etch approaches. In the etch-and-rinse approach, acid etching is performed on the dentine surface to remove the smear layer and preferentially demineralize the superficial layer of dentine exposing the collagen plexus which becomes infiltrated with the resin adhesive in a subsequent step. With the self-etch approach, acidic monomers undergo the etching together with a synchronized infiltration of the exposed collagen [14,69,70,71,72].

Endogenous collagenolytic enzymes, MMPs, are bound in mineralized dentine. The acidic treatment of the dentine surface activates MMPs present in dentine matrix in an inactive form, which become responsible for degradation of collagen in the hybrid layer. With the etch-and-rinse approach, the incomplete penetration of the collagen plexus of the hybrid layer leaves a denuded collagen layer at the bottom of the hybrid layer vulnerable to MMPs degradational activities [72]. Deterioration of resin dentine interfacial bonds can be due to degradation of the hybrid layer collagen fibrils, time dependent hydrolytic degradation of the hybrid layer resin component and the exogenous proteases including MMPs produced due to bacterial metabolic activities. Adverse clinical consequence of such deterioration includes increased hypersensitivity, recurrent caries, marginal discoloration, and development of reversible and irreversible pulpitis [14,69].

## 8. MMPs and Periodontal Tissues

MMPs play a significant role in the regulation and pathogenesis of periodontal diseases. Several medications and natural products could restore periodontal tissues inflammation through inhibition of MMPs and other related molecular cascades [73]. These findings are documented in clinical, in-vivo, and in-vitro studies. In this section, different pharmacological compounds that influenced periodontal tissues via modulation of matrix MMPs will be discussed.

Medications might have an impact on periodontal tissues via regulating MMPs. The antidiabetic medications, exenatide and sitagliptin, reduced the gingival expression of MMP-9 in ligature-induced periodontitis in rats without stabilizing the altered alveolar bone and collagen degradation [74]. Fluoxetine, a selective serotonin reuptake inhibitor, attenuated periodontal bone resorption and downregulated the activity of MMP-9 in the gingival tissues of Wistar rats with ligature-induced periodontal disease [75]. Celecoxib and omega-3 fatty acid treatments reduced gingival expression of MMP-8 and increased MMP-13 expressions in a Sprague-Dawley rat model of periodontitis by lipopolysaccharide [76]. Nifedipine, a calcium channel blocker, might increase the gene and protein expression of MMP-1 alone or in combination with interleukin-1alpha in human gingival fibroblasts [77]. Chlorhexidine mouthwash as an adjunctive therapy in patients with plaque-induced gingivitis had no effect on the levels of matrix metalloproteinase-8 in gingival crevicular fluid [78]. Chlorhexidine chip intraoral application following scaling and root planning lowered MMP-8 levels in the gingival crevicular fluid of chronic periodontitis patients [79]. Two synthesized bisphosphonic compounds and zoledronate, decreased the expression of MMP-9 and matrix metalloproteinases-14, while zoledronate increased MMP-8 expression in human gingival fibroblasts after exposure to lipopolysaccharide [80]. Another example of bisphosphonates, tiludronate, inhibited the activities of matrix metalloproteinase-1 and MMP-3 in human periodontal ligament cells in a dose dependent manner [81]. Batimastat inhibited the progression of periodontal tissue destruction in Sprague-Dawley rats through MMP inhibition [82]. S-nitrosoglutathione, a nitric oxide donor, reduced the MMP-1 and MMP-8 in the periodontium of Wistar rats in a ligature-induced periodontitis model [83]. Relaxin, a hormone that belongs to the insulin superfamily, increased MMP-1 and MMP-8 expression in human periodontal ligament cells [84]. A metal chelator namely phendione reduced the growth of Enterococcus faecalis in human root through inhibition of MMP-2 [85]. Moreover, excessive fluoride consumption increases MMP-2 expression in gingival and periodontal tissues of experimental rabbits [86]. Table 1 summarizes the MMPs that have been reported to contribute to gingival and periodontal lesions and wound healing, function, and associated diseases.

## 9. MMPs in Orthodontic Tooth Movement

Orthodontic tooth movement results from the forces exerted on a tooth that transmit pressure to periodontal ligaments (PDL) [88]. These mechanical stimuli trigger an inflammatory response in the periodontal tissues that elicit biochemical changes within the PDL leading to alveolar bone remodeling [89]. In addition, orthodontic tension as well as compression forces cause a continuous reorganization of the PDL extracellular matrix (ECM), which contributes to ECM deposition by secreting matrix proteins [90,91]. Expression of various proteolytic enzymes such as matrix-metalloproteinases (MMPs) are related to ECM protein degradation affecting PDL and alveolar bone remodeling [92]. MMPs-1, -2, -3, -7, -8, -12 and -13 are expressed in gingival crevicular fluid during orthodontic tooth movements [92]. Garlet et al. [93], showed increased MMP-1 expression levels in the PDL tissue at both the tension and compression areas with a significantly higher expression level at the compression zone. This indicates a potential higher importance of MMP-driven ECM protein degradation at the compression site.

Various studies involving in vivo models [89,94,95] using gingival crevicular fluid samples from healthy human orthodontic patients revealed increased levels of MMPs, including MMP-1, MMP-8 and MMP-13 collagenases within a broad time range after orthodontic treatment initiation at both the compression and tension zone. The increased MMP expression levels were partially higher at the compression zone [96]. In vitro studies [90,97] involving simulated orthodontic forces applied to cells isolated from the PDL revealed varying expression levels of MMPs and TIMPs, verifying a significant influence of mechanical forces.

An increased expression of MMP-8 and MMP-13 mRNA in the PDL of rats during active tooth movement has also been demonstrated [98]. Orthodontic tooth movement can be delayed or prevented in mice by the use of MMP inhibitors [99], which signifies their role in orthodontic tooth movement [96].

Table 2 summarizes the MMP groups and their involvement in dental and periodontal tissues and their role in orthodontic tooth movement.

## 10. MMP Inhibitors

MMPs can be inhibited by endogenous and exogenous inhibitors. Endogenous tissue inhibitors (TIMPs 1, 2, 3, 4) regulate and control MMP expression and function. Each TIMP has a specific gene regulation pattern, expression profile and binding affinity to specific MMPs [100]. TIMPs are present in the ECM in a soluble form, except for TIMP-3, which is bound to the ECM. All TIMPs inhibit MMPs through reversible blockage, forming 1:1 stoichiometric complexes [101].

Both MMPs and TIMPs have important roles in the maintenance of health and disease and their abnormal regulation has a relevant role in pathological conditions. Therefore, MMPs and TIMPs could be important biomarkers of disease [102]. For instance increased levels of MMP-8 and the MMP-8: TIMP-1 ratio in saliva and serum seem to be more pronounced in women with polycystic ovarian syndrome and they are potentiated by gingival inflammation [103]. TIMP1 might have a role in dental pulp inflammation [104]. Moreover, TIMP-1 is associated with acute apical periodontitis probably as a defense mechanism to avoid extensive destruction [105]. Accumulating evidence shows that both MMPs and TIMPs play a role in development, progress, and wound healing of apical periodontitis. However, more research is needed to elucidate the exact role of respective MMPs and TIMPs in the different stages of apical periodontitis and influences on severity of bone destruction and wound healing [106].

Designing exogenous MMP inhibitors traditionally aimed at displacing the zinc-bound water molecule by using zinc binding globulin. Current research focuses on fabricating MMP inhibitors that have selective specificity and ability to target MMPs with active site-directed potentials and structural identification capacity. Modern protein engineering technologies enabled the evolution of smart MMPs, responsive therapeutics and drug delivery vehicles.

Exogenous inhibitors in dentistry include multiple synthetic and natural compounds that can protect dentine and prevent the demineralization process via inhibition of the proteolytic activities of MMPs. Chlorhexidine, fluorinated products, indomethacin, tetracyclines, sodium trimetaphosphate, stannous chloride benzalkonium chloride, alcohols like ethanol, quaternary ammonium compounds [107], as well as other crosslinking and medicinal plants like green tea, grape seed extracts and curcumin are famous examples [10,23,102,103]. This section highlights the role of some chemical agents with MMP inhibiting effects and the therapeutic potential of pharmacological inhibition of MMPs in restorative dentistry.

### 10.1. Chlorhexidine

Chlorhexidine is extensively used in dental clinics as an antimicrobial agent to treat gingivitis and periodontitis. In addition, it prevents dental plaque and can be used as an adjunct to mechanical debridement. Chlorhexidine has marked effects as an exogenous inhibitor against matrix metalloproteases. It effectively and nonspecifically reduces collagen degradation by collagenolytic enzymes like MMPs and cysteine cathepsin [108].

It also provides inhibitory effects against MMPs in acidic environments produced by acid etching and dental caries. In an experimental study, pH sensitive nanocarriers of mesoporous silica loaded with chlorohexidine were incorporated in an experimental resin bonding agent to provide an MMP inhibiting effect in acidic microenvironments produced by acid etching and dental caries [109]. The controlled release of chlorohexidine at the dentine surface by adding clays to dentine bonding agents was found to improve durability of resin bonds to dentine [110]. Different studies and systematic reviews, indicate that chlorohexidine improves the long-term stability of resin bonds to dentine with some limitations concerning the test aging periods and the need for more supportive clinical data [111]. Osorio et al. also investigated whether the degradation of the dentine hybrid layer might be restricted by chlorhexidine digluconate following multiple demineralization techniques using phosphoric acid, EDTA or acidic monomers. They found that chlorhexidine has a partial inhibitory effect against MMPs in case of the acidic monomers, which was prolonged in comparison with phosphoric acid or EDTA [112]. Notably, the inhibitory activity of chlorhexidine, at concentrations of 0.5%, 1.0% and 2.0%, against MMPs were maintained after treating dentine powder with two-step self-etching primers [113].

### 10.2. Fluorinated Products

Fluorinated products are a useful tool in dentistry to prevent dental caries. Studies showed that they have MMP inhibitory effects. It was suggested that fluoride, in the form of sodium fluoride, might prevent dental caries through inhibition of salivary and purified human gelatinases MMP-2 and MMP-9 [51]. In contrast, it was reported that sodium fluoride might show low efficiency as a direct inhibitor of dentine matrix-bound matrix metalloproteinases [114]. Another study by the same research group demonstrated that potassium fluoride might inhibit the proteolytic properties of dentine matrix-bound cysteine cathepsins without a visible efficacy against dentine MMP activity [115]. Treatment of dentine with sodium trimetaphosphate, a synthetic compound that reduces dentine demineralization, inhibited MMP-2 and MMP-9 activities particularly at 1.5% concentration [116]. Other synthetic compounds that preserve dentine mineralization via MMP-2 and MMP-9 include stannous chloride and stannous fluoride [117].

Dentifrices that contain MMP inhibitors including sodium fluoride, green tea extract, or chlorhexidine digluconate can markedly decrease dentine loss [49], preserve the surface properties of eroded dentine specimens and counteract dentine abrasions and erosions [49].

### 10.3. Tetracyclines

Tetracyclines have innate MMP inhibitory capacity. Doxycycline is indicated for use in periodontal disease and is the only collagenase inhibitor approved by the US Food and Drug Administration for any human disease [118].

Chemically modified tetracycline-3 showed preservative ability against the progression and prevalence of dentine caries in rats [44]. Oliveira et al., 2016 reported that pretreatment with doxycycline either as acidic or neutral solutions had no effect on bond strength of dentine adhesive [119]. Moreover, encapsulated doxycycline, as a MMP inhibitor, might improve the durability and performance of hybrid layers in adhesively bonded resin used in restorative dentistry [120]. Inhibition of MMP activities using chemically modified tetracycline-3 lowered the organic bone matrix degradation in rats and resulted in reduced tooth movement [121].

Other synthetic inhibitors of matrix metalloproteinases such as galardin were assessed to determine their inhibitory effects in dentine [122]. Indomethacin was also assessed to evaluate its inhibitory effect against the enzymatic activity of MMPs in dentine. In this context, indomethacin-treated dentine samples had hindered enzymatic activities [123].

## 11. Concluding Remarks

Understanding the role and biofunctional aspects of MMPs constitutes an integral part in figuring out the micromolecular basis of health and disease processes. This can open the door for future paradigm shifts in diagnostic and therapeutic strategies. The active and dynamic participation of MMPs in developmental, degradational and pathological processes in dental tissues is increasingly drawing the attention of researchers. Research endeavors highlight the role of MMPs in the formative amelogenesis and dentinogenesis as well as in degradation of collagen in the hybrid layer, progress of dental caries, pulp and periapical inflammation, in addition to the healing of wounds of the dentine-pulp organ. Nevertheless, the role of MMPs in periodontal pathology and remodeling during orthodontic tooth movements is evident. Ongoing research should continue to develop clinically effective MMP inhibitors with sustained potency to protect dentine matrix and provide adequate therapy for dentine caries, preserve the collagen hybrid layer, and maintain the long-term integrity of resin-dentine bonds, and facilitate remineralization, repair and regeneration of dental tissues. Moreover, future studies should continue to validate the suitability of using MMPs as diagnostic and prognostic biomarkers in dental caries, pulp, and periodontal lesions.

## Figures and Tables

**Table 1:** MMPS in periodontal lesions modified from the work of Ionut Luchian et al. (Luchian I, Goriuc A, Sandu D, Covasa M. The Role of Matrix Metalloproteinases (MMP-8, MMP-9, MMP-13) in Periodontal and Peri-Implant Pathological Processes. *Int J Mol Sci.* **2022**, *23*, 1806. https://doi.org/10.3390/cells9051313. PMID: 35163727; PMCID: PMC8837018. (MDPI open access source) [87].

Type of MMP	Name	Substrate	Production	Physiological Function	Associated Diseases
**MMP-1**	Collagenase 1/Interstitial Collagenase/Fibroblast Collagenase	Collagen I, II, III, VII, VIII, X, XI, Gelatin, Fibronectin, Aggrecan, Entactin, Tenascin, Ovostatin, Casein	Fibroblast, Keratinocytes, Endothelial cells, Macrophages, Osteoblast, Chondrocytes, Platelet	Wound healing, re-epithelialization, cell proliferation, keratinocyte migration	Periodontitis, rheumatoid arthritis, atherosclerosis, fibrosis, autoimmune disease, cancer
**MMP-2**	Gelatinase A/72-kDa type IV collagenase	Collagen, Elastin, Endothelin, Fibroblast growth factor, MMP-9, MMP-13, Plasminogen, and TGF-β,	Cardiomyocytes, Fibroblasts, and Myofibroblasts.	Neovascularization, Angiogenesis, Promoting and inhibiting Inflammation,	Cancer, asthma, lung diseases,
**MMP-8**	Collagenase 2/Neutrophil Collagenase	Collagen I, II, III, Fibronectin, Aggrecan, Ovostatin	Chondrocytes, Endothelial cell, Macrophages, Smooth muscle cell	Periodontal tissue turnover, Anti-inflammatory activity, Wound healing	Periodontitis, rheumatoid arthritis, asthma, cancer
**MMP-9**	Gelatinase B/92-kDa type IV collagenase	Gelatin, Type V collagen, Laminin, Fibronectin	Neutrophils, Eosinophils, Epithelial cells	Wound healing, embryo implantation, neovascularization, immune cells function, tissue remodeling	Arthritis, metastasis, pulmonary disease, infections, cardiovascular disease, periodontal disease
**MMP-12**	Macrophage elastase	Elastin, Laminin, Fibronectin, Vitronectin, Type IV collagen	Endothelial cells, Neutrophils, Fibroblasts, T-cells, Myocytes, Macrophages,	degrade extracellular matrix component	Emphysema, arthritis, cancer, periodontal disease
**MMP-13**	Collagenase 3	Collagen I, II, III, IV, IX, X, XIV, Fibronectin, Laminin, Gelatin, Aggrecan, Plasminogen, Osteonectin	Epithelial cell, Neuronal cell, Connective tissue (Cartilage and Bone)	Osteoclastic activation, anti-inflammatory activity	Periodontitis, osteoarthritis, liver fibrosis, cancer

**Table 2:** MMP groups and their roles in dental and periodontal tissues and in orthodontic tooth movement. (MMP groups were used from Jain A, Bahuguna R. Role of matrix metalloproteinases in dental caries, pulp, and periapical inflammation: An overview. *J. Oral Biol. Craniofac. Res.* **2015**, *5*, 212–218. https://doi.org/10.1016/j.jobcr.2015.06.015. PMID: 26605147; PMCID: PMC4623218 with a permission to use [4].

MMPs Groups	Enamel	Dentine	Pulp and Periapical Lesions	Periodontal Ligament	Dental Caries	Orthodontic Tooth Movement
**1. Collagenases** MMP-1 MMP-8 MMP-13	MMP-13 mucosal penetration during tooth eruption	MMP-8 and MMP-13 Dentinogenesis MMP-8 reparative dentine formation	MMP-1, -8, -13 reparative dentine formation MMP-1 acute pulpitis	MMP-1 periodontitis MMP-8 gingivitis MMP-13 periodontitis	MMP-1, 8 pathogenesis of caries	MMP-1, -8, -13 expressed in crevicular fluid during orthodontic treatment
**2. Gelatinases** MMP-2 MMP-9	MMP-2, MMP-9 mucosal penetration during tooth eruption	MMP-2, 9 dentinogenesis	MMP-9 reparative dentine formation MMP-2 acute pulpitis MMP-2, -9 progression of apical periodontitis MMP-9 alveolar bone resorption	MMP-9 gingivitis	MMP-2, 9 pathogenesis of caries	MMP-2 expressed in crevicular fluid during orthodontic treatment
**3. Stromelysins** MMP-3 MMP-10 MMP-11 MMP-12	MMP-3 dentine mineralization		MMP-3 reparative dentine formation		MMP-3 pathogenesis of caries	MMP-3, -12 expressed in crevicular fluid during orthodontic treatment
**4. Matrilysins** MMP-7 MMP-26						MMP-7 expressed in crevicular fluid during orthodontic treatment
**5. MT-MMPs** **(Membrane type)** MMPs-14(MT1-MMP) MMPs-15(MT2-MMP) MMPs-16(MT3-MMP) MMPs-17(MT4-MMP) MMPs-24(MT5-MMP) MMPs-25(MT6-MMP)	MMPs-14 dentinogenesis MT1-MMPs and TIMP-2 correlate to the rate of tooth eruption				MMP-16 pathogenesis of caries	
**6. Other MMPs** MMPs-18 MMP-19 MMPs20(enamelysin) MMPs21 MMPs-23 MMPs-27 MMPs-28	MMPs-19 dentinogenesis MMPs-20 (amelogenesis and dentinogenesis)		MMP-20 reparative dentine formation		MMP-20 pathogenesis of caries	

## Data Availability

Not applicable.
